# RyR2 Gain-of-Function Increases Ca^2+^ Spark and Ca^2+^ Wave Activity in Vascular Smooth Muscle Cells

**DOI:** 10.3390/ijms27219636

**Published:** 2026-10-28

**Authors:** Daniele Teixeira Alves, Qidi Zhai, Liming Lu, Zhugang Chu, Dmitry Tsvetkov, S. R. Wayne Chen, Maik Gollasch, Mario Kassmann

**Affiliations:** 1 Department of Internal Medicine and Geriatrics, University Medicine Greifswald, 17475 Greifswald, Germany; qidi.zhai@med.uni-greifswald.de (Q.Z.); lu.liming@med.uni-greifswald.de (L.L.); chuzhugang898@gmail.com (Z.C.); dmitry.tsvetkov@med.uni-greifswald.de (D.T.); maik.gollasch@med.uni-greifswald.de (M.G.); 2 DZHK ( German Centre for Cardiovascular Research), Partner Site Greifswald, 17475 Greifswald, Germany; 3 Libin Cardiovascular Institute of Alberta, Department of Physiology and Pharmacology, University of Calgary, Calgary, AB T2N 4Z6, Canada; swchen@ucalgary.ca

**Keywords:** Ca^2+^ waves, ryanodine receptor 2, vascular smooth muscle cells, RyR2^R4496C^ mutation, mesenteric artery, ageing, metabolic stress

## Abstract

Ca^2+^ sparks in vascular smooth muscle cells (VSMCs) regulate vascular function, and their dysregulation with ageing may contribute to vascular disease. These localized Ca^2+^ signals originate from ryanodine receptors (RyRs) and can initiate propagating Ca^2+^ waves that modulate vascular tone. However, it is still unclear which RyR isoform (RyR1, RyR2, or RyR3) primarily drives these Ca^2+^ signals in arterial VSMCs, as all three are present. The RyR2 contribution to vascular Ca^2+^ signaling was analyzed using Ca^2+^ sparks and waves in arterial VSMCs isolated from mice carrying the RyR2^R4496C^ mutation. Intracellular Ca^2+^ signals were recorded using a fluorescent Ca^2+^ indicator in VSMCs from both young and older mice. Caffeine-evoked Ca^2+^ responses and contractions were measured to assess RyR-mediated SR Ca^2+^ release and vascular reactivity. VSMCs from young RyR2^R4496C^ mice exhibited a significantly higher frequency of Ca^2+^ sparks and a greater percentage of cells displaying Ca^2+^ sparks and propagating Ca^2+^ waves compared with controls. The RyR2^R4496C^ mutation enhanced RyR2 sensitivity to caffeine, resulting in larger increases in intracellular [Ca^2+^]_i_ and arterial contractions in older mice. These findings demonstrate that RyR2 gain-of-function increases Ca^2+^ spark and Ca^2+^ wave activity in VSMCs, supporting a role for RyR2 in the regulation of local and propagating Ca^2+^ signals. Dysregulated RyR2 activity may therefore contribute to altered vascular Ca^2+^ signaling and could represent a potential mechanistic link to vascular dysfunction associated with ageing and metabolic stress.

## 1. Introduction

Ageing is associated with progressive vascular dysfunction that contributes to increased cardiovascular risk, frailty, and reduced physiological resilience in older populations [1]. Conditions commonly associated with ageing, such as diabetes and diet-induced metabolic disorders, further disrupt vascular homeostasis and contribute to impaired arterial function [2,3]. Nutritional and metabolic factors represent important modulators of vascular ageing, influencing vascular health, functional decline, and physiological resilience in older individuals [4].

Intracellular Ca^2+^ signaling is a key regulator of vascular smooth muscle cell (VSMC) function and arterial tone. In resistance artery VSMCs, Ca^2+^ influx through voltage-dependent Ca^2+^ channels activates RyRs in the sarcoplasmic reticulum (SR), resulting in spatially restricted, transient Ca^2+^ release events termed Ca^2+^ sparks [5,6]. These Ca^2+^ sparks activate large-conductance Ca^2+^-activated K^+^ (BK_Ca_) channels, generating spontaneous transient outward currents (STOCs) that hyperpolarize the membrane and reduce Ca^2+^ influx through L-type Ca_v_1.2 channels, thereby limiting arterial constriction [5,7,8]. Under certain conditions, Ca^2+^ sparks can recruit neighboring release sites and generate propagating Ca^2+^ waves. These Ca^2+^ waves can propagate throughout entire cells [9]. However, in contrast to the intestine [10] and upper urinary tract smooth muscle tissues [11], where specialized pacemaker cells initiate coordinated peristaltic activity, comparable pacemaker cells are absent in the arterial vascular system. Instead, resistance arteries develop myogenic tone, which is regulated by factors such as transmural pressure, blood flow, and intrinsic vascular smooth muscle signaling [12].

RyRs exist in three isoforms (RyR1, RyR2, and RyR3), all of which are expressed in arterial vascular smooth muscle [13,14]. However, the specific RyR isoform responsible for Ca^2+^ spark generation remains incompletely understood. RyRs assemble as homotetrameric channels that cluster within the SR membrane. The coordinated opening of multiple RyRs—estimated to involve approximately 10–200 channels—is required to produce a Ca^2+^ spark [6,15]. Because different RyR isoforms may coexist within shared channel clusters, loss-of-function approaches may disrupt the entire RyR signaling network rather than reveal the contribution of individual isoforms. Therefore, isoform-specific gain-of-function strategies are required to define the RyR isoform responsible for Ca^2+^ spark generation.

Consistent with this concept, previous genetic studies have revealed distinct and isoform-specific roles of RyR channels in regulating intracellular Ca^2+^ signaling. Genetic ablation of RyR3 increased Ca^2+^ spark activity in arterial VSMCs [13], whereas conditional deletion of RyR2 abolished Ca^2+^ sparks [14]. In addition, heterozygous or homozygous deletion of RyR1 reduced Ca^2+^ spark activity in the aorta, pulmonary arteries and portal vein [16,17]. Together, these findings indicate that the functional contribution of the different RyR isoforms to vascular Ca^2+^ signaling is complex and not yet fully resolved.

The RyR2 mutation R4496C, the murine equivalent of the human R4497C variant, is associated with catecholaminergic polymorphic ventricular tachycardia (CPVT), an inherited arrhythmogenic disorder [18]. This mutation confers a gain-of-function phenotype characterized by an increased open probability of the RyR2 channel and enhanced spontaneous Ca^2+^ release [19]. While these effects have been extensively studied in cardiomyocytes, their impact on Ca^2+^ signaling in vascular smooth muscle is unknown.

In the present study, we investigated whether enhanced RyR2 activity influences Ca^2+^ spark and Ca^2+^ wave generation in arterial VSMCs using mice carrying the RyR2^R4496C^ gain-of-function mutation. By analyzing Ca^2+^ signaling in mesenteric artery VSMCs from young and older mice, we aimed to clarify the contribution of RyR2 to vascular Ca^2+^ signaling and its potential role in vascular dysfunction associated with ageing and metabolic stress.

## 2. Results

Additional results are presented in the Supplementary Materials.

### 2.1. Ca^2+^ Sparks and Waves in Young VSMCs

**Figure 1:**
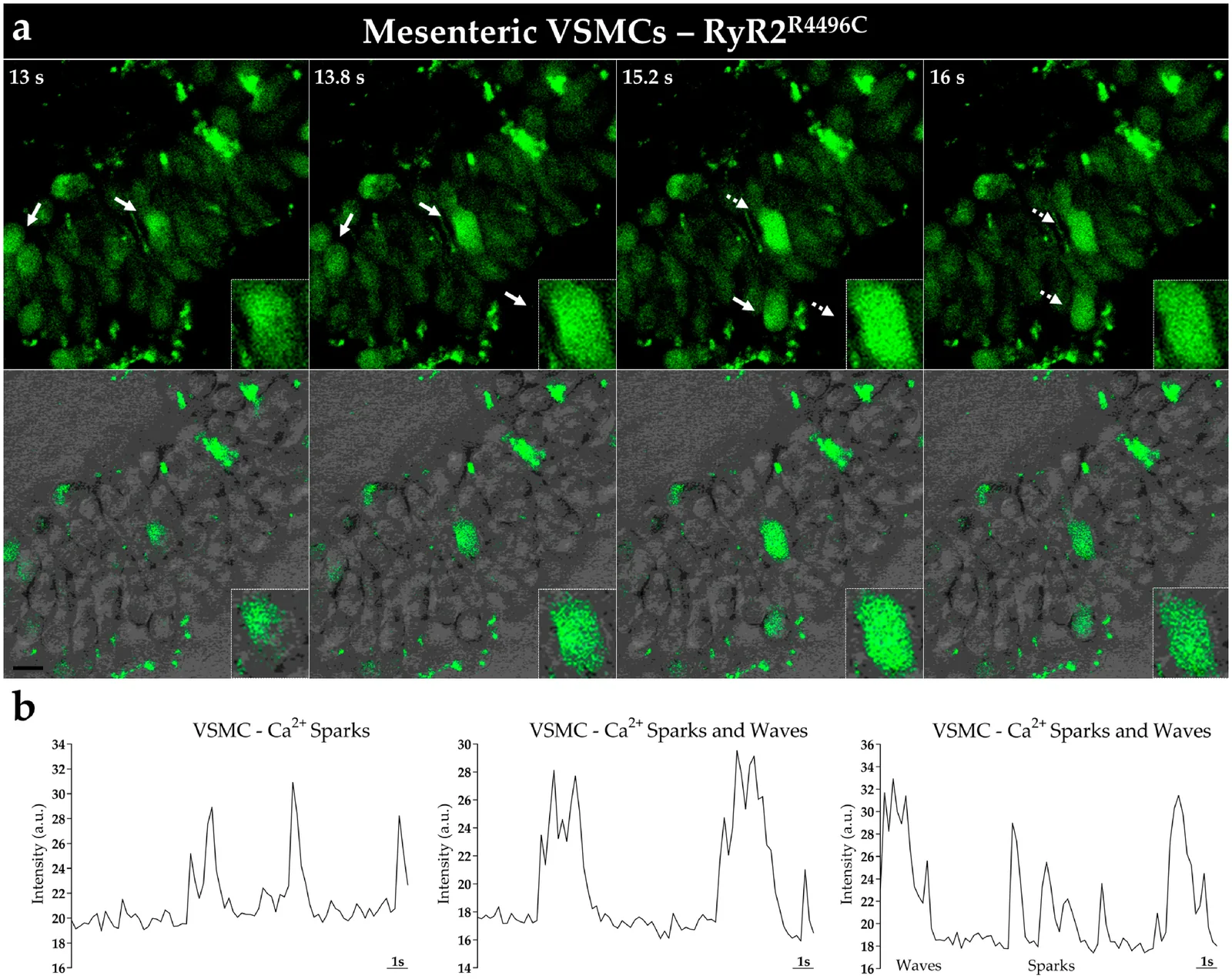
Original recordings of Ca^2+^ sparks and Ca^2+^ waves in in situ clustered VSMCs isolated from young mice carrying the RyR2R^4496C^ mutation. (**a**) Ca^2+^ sparks followed by propagating Ca^2+^ waves, detected using the fluorescent Ca^2+^ indicator Fluo-4 AM (green). The white arrows indicate Ca^2+^ sparks, while the dashed arrows highlight Ca^2+^ waves. Enlarged images are shown at the bottom right. The temporal intervals between the displayed recordings are 800 ms, 1400 ms, and 800 ms. A clearer view of cell morphology together with the corresponding fluorescent signals is presented below using the T-PMT channel. Scale bar = 10 µm. (**b**) Representative time course of Ca^2+^ fluorescence changes showing cell-by-cell analysis of Ca^2+^ sparks and Ca^2+^ waves.

We first examined Ca^2+^ sparks and propagating Ca^2+^ waves in VSMCs isolated from young control mice and age-matched mice carrying the RyR2^R4496C^ mutation. Using the fluorescent Ca^2+^ indicator Fluo-4 AM, we detected spontaneous Ca^2+^ sparks that frequently triggered propagating Ca^2+^ waves within individual VSMCs (Figure 1a). High-resolution recordings revealed localized Ca^2+^ release events that initiated wave-like Ca^2+^ propagation along the cell. Cell morphology and corresponding fluorescent Ca^2+^ signals were visualized using the T-PMT channel. Cell-by-cell analysis confirmed the presence of Ca^2+^ sparks and waves in individual VSMCs (Figure 1b).

**Figure 2:**
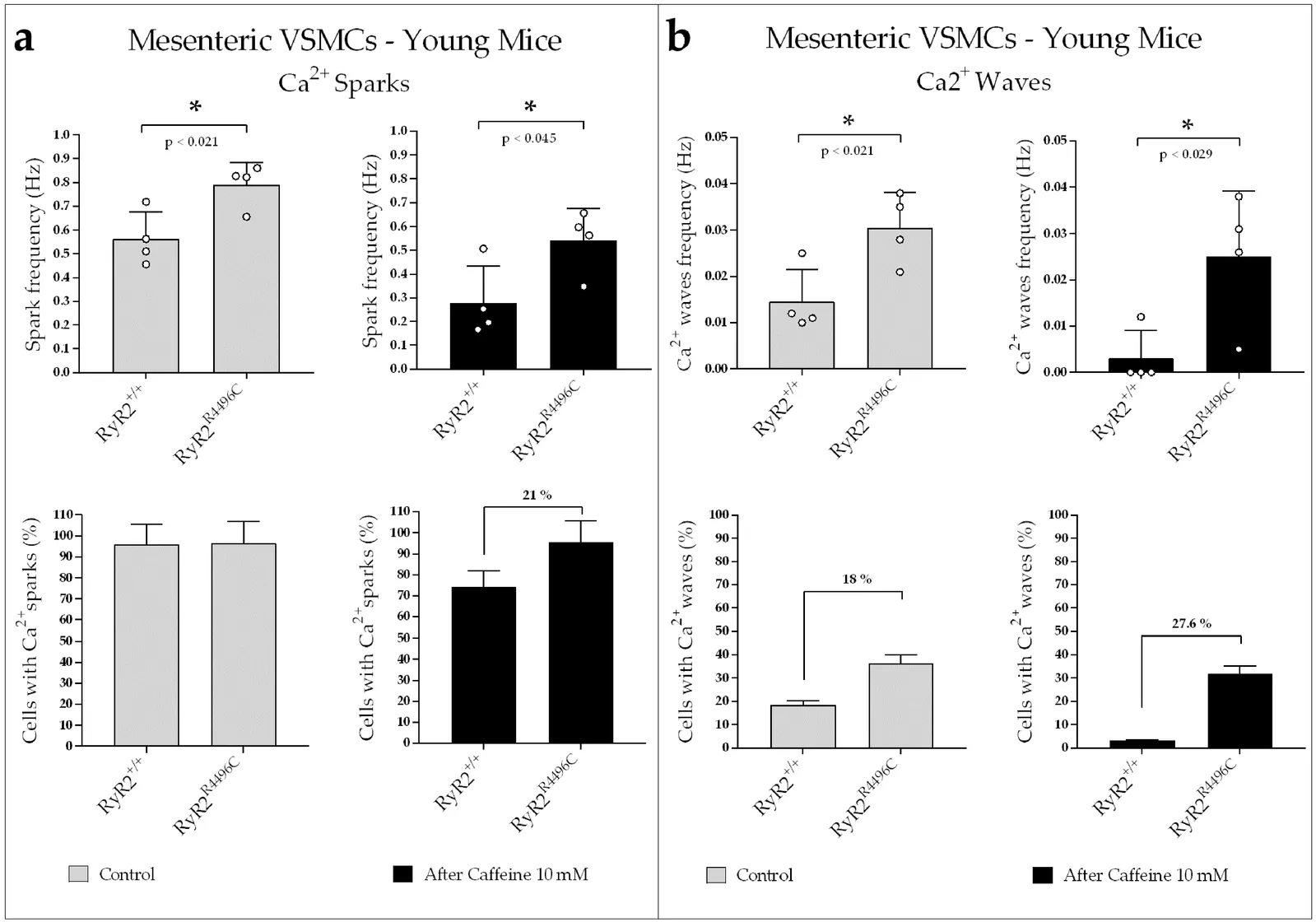
The R4496C mutation increases the frequency of Ca^2+^ sparks and propagating Ca^2+^ waves in clustered VSMCs isolated from young mice. (**a**) Frequency and percentage of Ca^2+^ sparks in RyR2^+/+^ and RyR2^R4496C^ VSMCs isolated from young mice, measured before and 10 min after depleting SR Ca^2+^ by caffeine. (**b**) Frequency and percentage of Ca^2+^ waves in RyR2^+/+^ and RyR2^R4496C^ VSMCs isolated from young mice, measured before and 10 min after depleting SR Ca^2+^ by caffeine. Values are means ± SEM. * *p* < 0.05 vs. RyR2^+/+^ (Student’s *t*-test). The percentage of Ca^2+^ sparks and waves is expressed as the proportion of cells exhibiting sparks and waves. Data were obtained from RyR2^+/+^ mice (*n* = 4; 20–26 VSMCs recorded and analyzed per mouse) and RyR2^R4496C^ mice (*n* = 4; 20–25 VSMCs recorded and analyzed per mouse).

Next, we quantified the frequency and occurrence of Ca^2+^ sparks and Ca^2+^ waves in VSMCs isolated from young RyR2^R4496C^ mice and age-matched RyR2^+/+^ controls. We found that the R4496C mutation significantly increased both the frequency of Ca^2+^ sparks and the percentage of cells exhibiting these events compared with RyR2^+/+^ VSMCs (Figure 2a). Similarly, the mutation significantly increased the frequency and percentage of cells displaying propagating Ca^2+^ waves (Figure 2b). These effects were observed under basal conditions and after stimulation with 10 mM caffeine.

**Figure 3:**
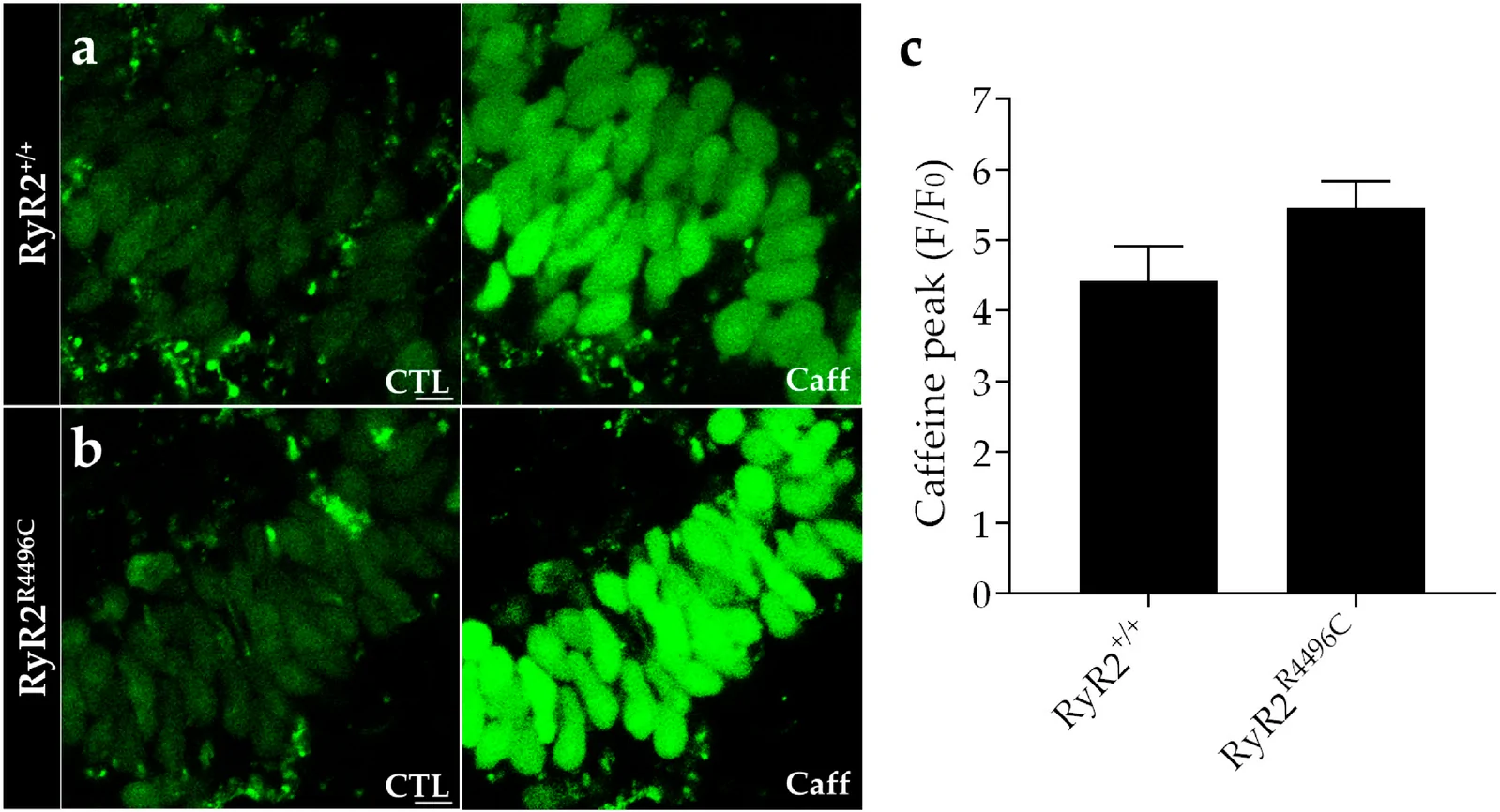
Effects of the RyR2 R4496C mutation on caffeine sensitivity in clustered VSMCs isolated from young mice. Original images showing intracellular calcium increases ([Ca^2+^]_i_) in response to 10 mM caffeine (**a**,**b**). Scale bar = 10 µm. Summary data (**c**). Values are means ± SEM. Data were obtained from RyR2^+/+^ (*n* = 4 mice; 20–26 VSMCs analyzed per mouse) and RyR2^R4496C^ mice (*n* = 4 mice; 20–25 VSMCs analyzed per mouse).

We then examined whether the RyR2^R4496C^ mutation alters the sensitivity of intracellular Ca^2+^ release to caffeine. Acute application of 10 mM caffeine induced increases in intracellular Ca^2+^ concentration ([Ca^2+^]_i_) in both RyR2^+/+^ and RyR2^R4496C^ VSMCs (Figure 3a,b). However, no difference in the caffeine-induced increase in [Ca^2+^]_i_ was observed between cells expressing the RyR2^R4496C^ mutation and control cells (Figure 3c).

### 2.2. Ca^2+^ Sparks and Waves in Older VSMCs

**Figure 4:**
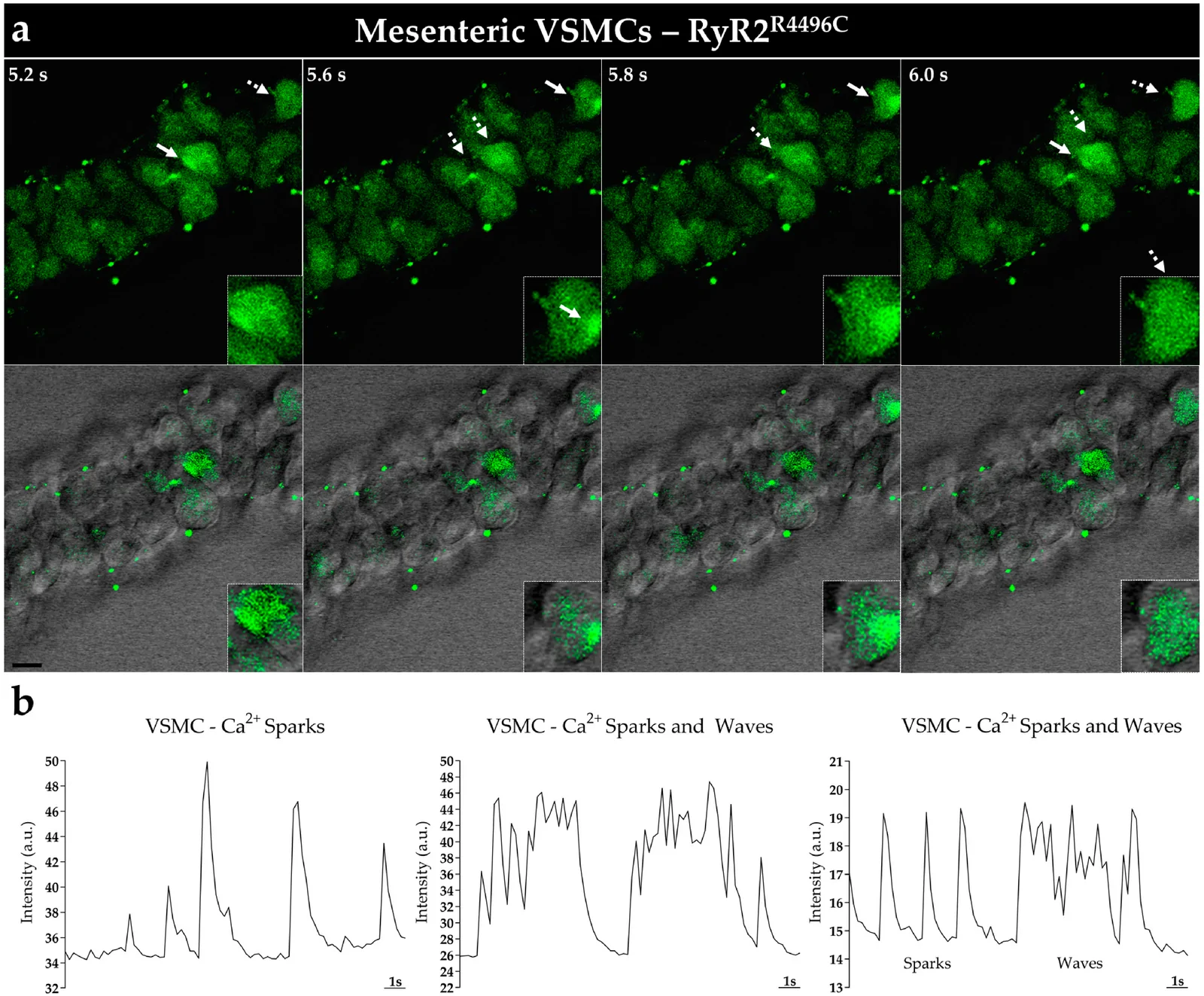
Original recordings of Ca^2+^ sparks and Ca^2+^ waves in clustered VSMCs isolated from older mice carrying the RyR2^R4496C^ mutation. (**a**) Ca^2+^ sparks followed by propagating Ca^2+^ waves, detected using the fluorescent Ca^2+^ indicator Fluo-4 AM (green). The white arrows indicate Ca^2+^ sparks, while the dashed arrows highlight Ca^2+^ waves. Enlarged images are shown at the bottom right. The temporal intervals between the displayed recordings are 400 ms, 200 ms, and 200 ms. A clearer view of cell morphology together with the corresponding fluorescent signals is presented below using the T-PMT channel. Scale bar = 10 µm. (**b**) Representative time course of Ca^2+^ fluorescence changes showing cell-by-cell analysis of Ca^2+^ sparks and Ca^2+^ waves.

To determine whether RyR2-dependent alterations in vascular Ca^2+^ signaling persist during ageing, we next analyzed Ca^2+^ sparks and Ca^2+^ waves in VSMCs isolated from older RyR2^+/+^ and RyR2^R4496C^ mice. As observed in young animals, spontaneous Ca^2+^ sparks were detected in older VSMCs and initiated propagating Ca^2+^ waves. (Figure 4a). Cell-by-cell analysis confirmed the presence of these events in individual cells (Figure 4b).

**Figure 5:**
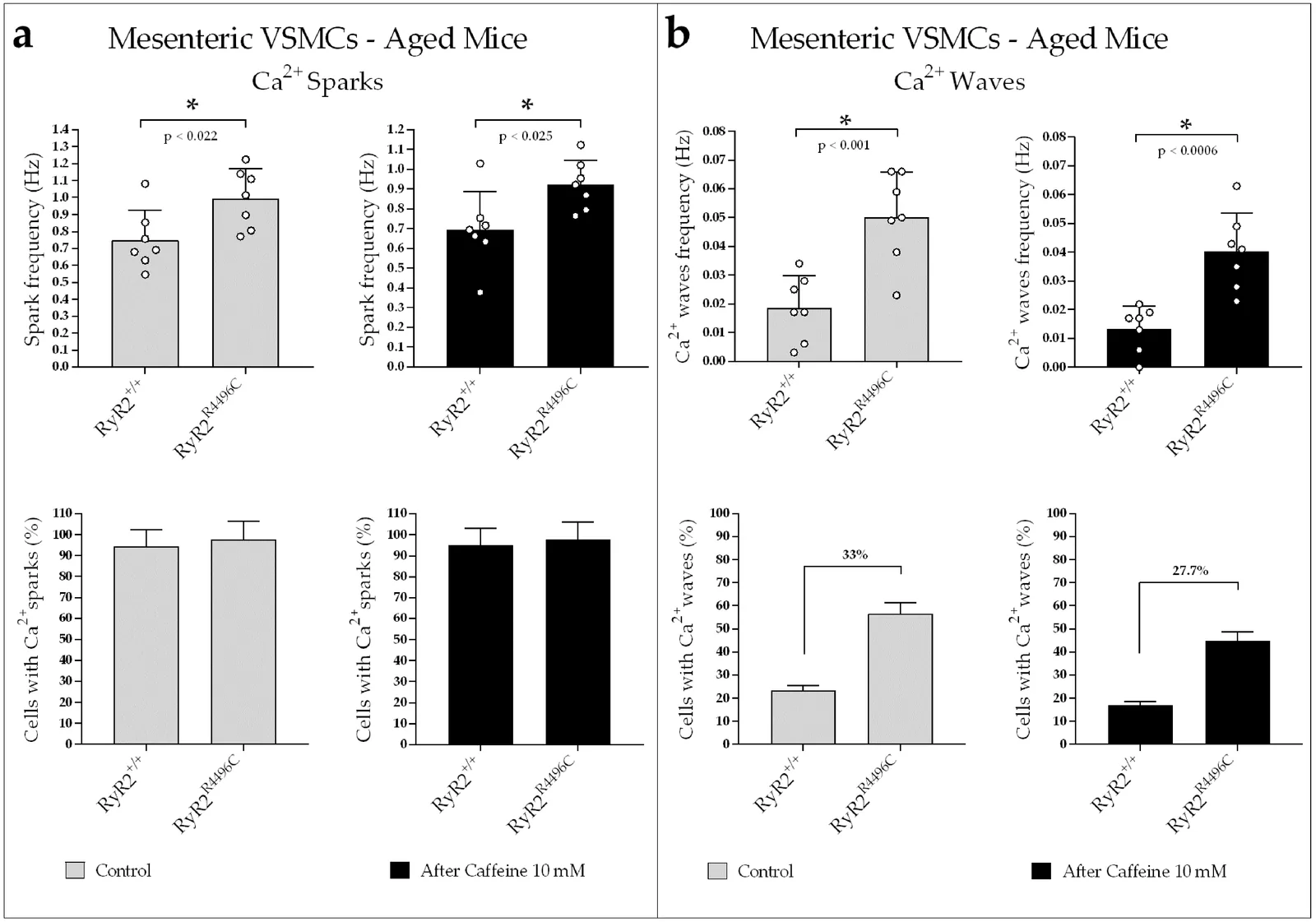
The R4496C mutation increases the frequency of Ca^2+^ sparks and propagating Ca^2+^ waves in clustered VSMCs isolated from older mice. (**a**) Frequency and percentage of Ca^2+^ sparks in RyR2^+/+^ and RyR2^R4496C^ VSMCs isolated from older mice, measured before and 10 min after depleting SR Ca^2+^ by caffeine. (**b**) Frequency and percentage of Ca^2+^ waves in RyR2^+/+^ and RyR2^R4496C^ VSMCs isolated from older mice, measured before and 10 min after depleting SR Ca^2+^ by caffeine. Values are means ± SEM. * *p* < 0.05 vs. RyR2^+/+^ (Student’s *t*-test). The percentage of Ca^2+^ sparks and waves is expressed as the proportion of cells exhibiting sparks and waves. Data were obtained from older RyR2^+/+^ (*n* = 7 mice; 20–21 VSMCs analyzed per mouse) and RyR2^R4496C^ mice (*n* = 7 mice; 18–20 VSMCs analyzed per mouse).

Quantitative analysis revealed that the R4496C mutation significantly increased the frequency of Ca^2+^ sparks and the proportion of cells displaying sparks in VSMCs isolated from older mice (Figure 5a). In addition, the mutation increased the frequency and percentage of cells exhibiting propagating Ca^2+^ waves compared with RyR2^+/+^ controls (Figure 5b).

### 2.3. Contribution of Ca^2+^ Influx Pathways to RyR2^R4496C^ Ca^2+^ Sparks

To further investigate whether Ca^2+^ influx pathways contribute to the increased Ca^2+^ spark activity in RyR2^R4496C^ VSMCs, we next examined the sensitivity of these events to inhibitors targeting major Ca^2+^ entry pathways. We previously showed that Ca^2+^ influx through Ca_v_1.2 L-type Ca^2+^ channels represents the predominant pathway [20], whereas Ca_v_3.2 T-type Ca^2+^ channels and non-selective Ca^2+^-permeable (TRP) cation channels play little or no role in this process [21]. Blockade of L-type Ca^2+^ channels nearly abolished RyR2^R4496C^-dependent Ca^2+^ sparks, whereas inhibition of Ca_v_3.2 T-type Ca^2+^ channels with Ni^2+^ or TRP channels with Gd^3+^ was largely ineffective (Supplementary Figure S1). These findings demonstrate that the same hierarchy of Ca^2+^ influx pathways, dominated by L-type Ca^2+^ channels, regulates Ca^2+^ spark activity mediated by mutant RyR2^R4496C^ channels in mesenteric artery VSMCs.

**Figure 6:**
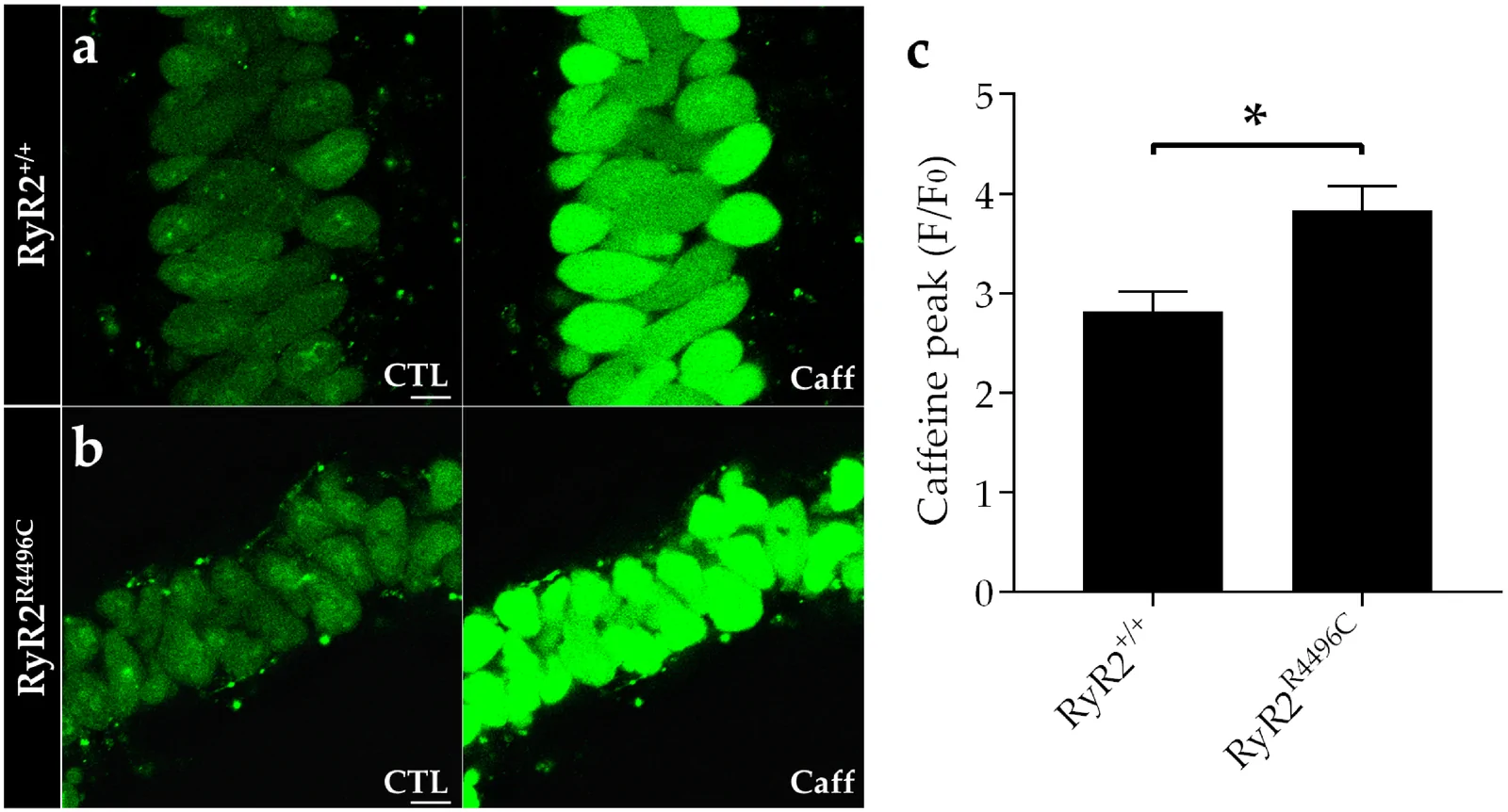
The R4496C mutation enhances RyR2 sensitivity to caffeine in clustered VSMCs isolated from older mice. Original images showing intracellular calcium increases ([Ca^2+^]_i_) in response to 10 mM caffeine (**a**,**b**). Scale bar = 10 µm. Summary data (**c**). Values are means ± SEM. * *p* < 0.05. Data were obtained from RyR2^+/+^ (*n* = 7 mice; 20–21 VSMCs analyzed per mouse) and RyR2^R4496C^ mice (*n* = 7 mice; 18–20 VSMCs analyzed per mouse).

We further assessed caffeine-induced Ca^2+^ release in older VSMCs. Application of 10 mM caffeine increased [Ca^2+^]_i_ levels in both genotypes (Figure 6a,b). The caffeine-induced [Ca^2+^]^i^ peak was significantly larger in RyR2^R4496C^ than in RyR2^+/+^ VSMCs (Figure 6c).

### 2.4. Vascular Function

**Figure 7:**
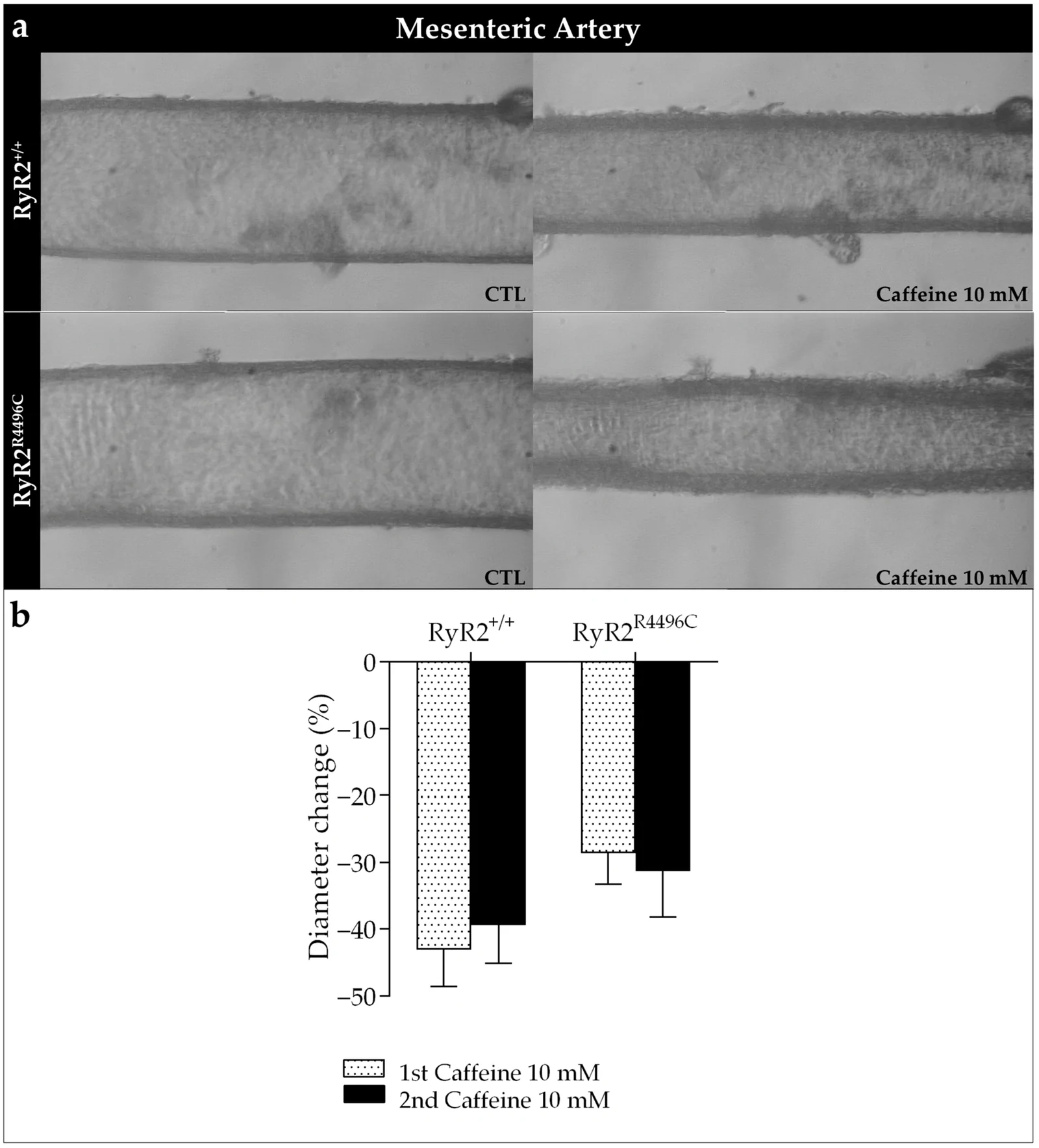
RyR2-mediated caffeine-induced vasoconstriction in mesenteric arteries from young RyR2^R4496C^ mice. Representative images of mesenteric arteries from RyR2^+/+^ (upper panels) and RyR2^R4496C^ mice (lower panels) before and during caffeine-induced contraction using pressure myography (**a**). Caffeine-induced vasoconstriction did not differ between young RyR2^R4496C^ and RyR2^+/+^ mice (**b**) (Sidak’s multiple comparisons test). Values are means ± SEM. *n* = 5 mice per group; one mesenteric artery was analyzed per mouse.

**Figure 8:**
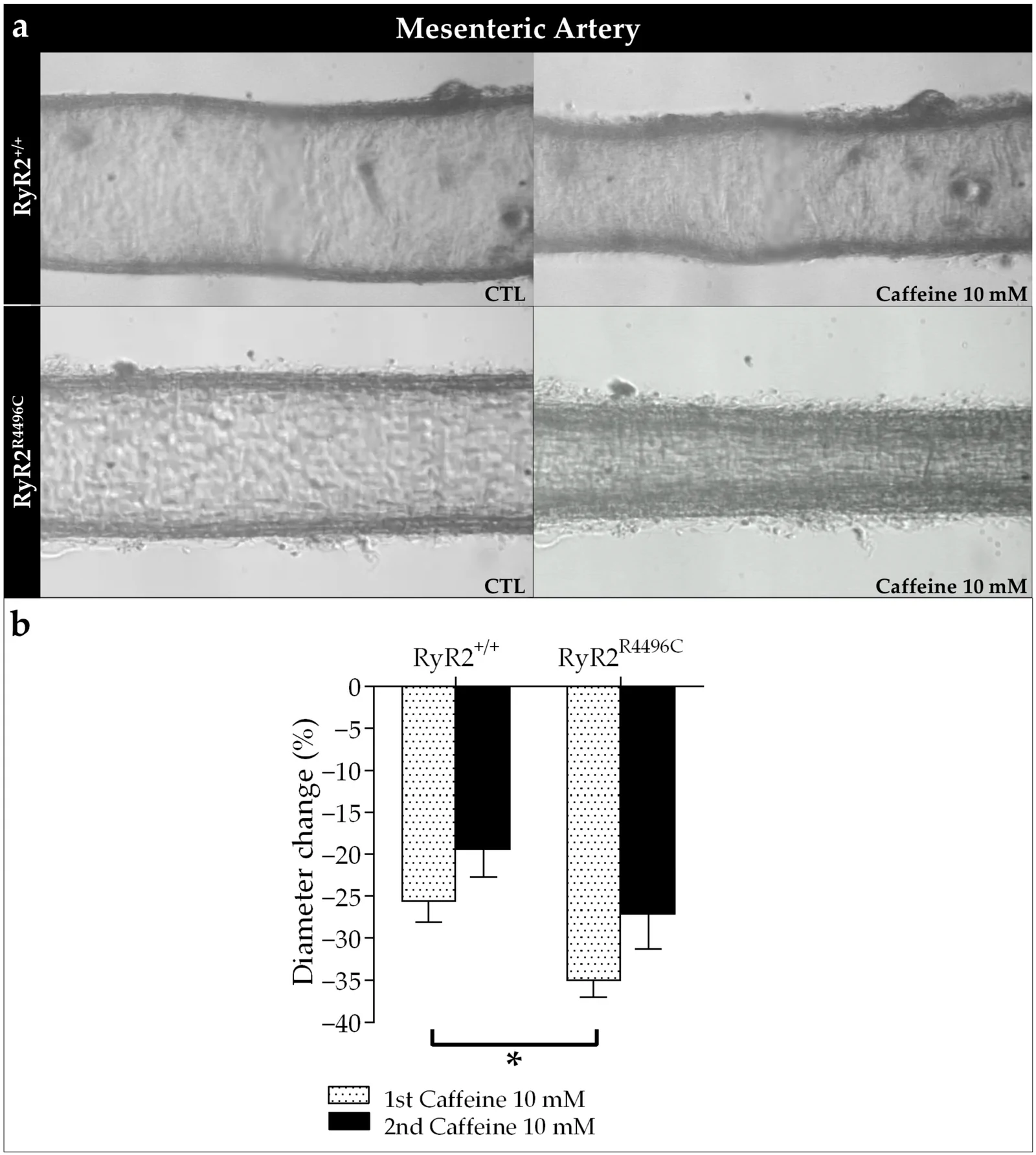
Enhanced RyR2-mediated caffeine-induced vasoconstriction in mesenteric arteries from older RyR2^R4496C^ mice. Representative images of mesenteric arteries from RyR2^+/+^ (**upper panels**) and RyR2^R4496C^ mice (**lower panels**) before and during caffeine-induced contraction using pressure myography (**a**). Caffeine-induced vasoconstriction was significantly greater in older RyR2^R4496C^ than in RyR2^+/+^ mice (**b**). Values are means ± SEM. * *p* < 0.05 vs. RyR2^+/+^ (Sidak’s multiple comparisons test). *n* = 6 mice per group; one mesenteric artery was analyzed per mouse.

Finally, we investigated whether the RyR2^R4496C^ mutation alters vascular function at the tissue level. To evaluate the functional consequences of altered RyR2-mediated Ca^2+^ signaling, caffeine-induced contractions of isolated mesenteric arteries were assessed using pressure myography. Mesenteric arteries from young RyR2^R4496C^ mice showed similar caffeine-induced vasoconstriction compared with RyR2^+/+^ controls (Figure 7), whereas vasoconstriction was significantly enhanced in older RyR2^R4496C^ arteries (Figure 8), consistent with increased RyR2-mediated global SR Ca^2+^ release in intact vessels. Similar caffeine-induced contractions in young mice were confirmed by wire myography (Supplementary Figure S2).

## 3. Discussion

The present study demonstrates that RyR2 gain-of-function increases Ca^2+^ spark and Ca^2+^ wave activity in arterial vascular smooth muscle cells. Using VSMCs isolated from mice carrying the gain-of-function RyR2^R4496C^ mutation, we show that spontaneous Ca^2+^ spark frequency and the occurrence of propagating Ca^2+^ waves are increased in both young and older animals. In addition, older RyR2^R4496C^ VSMCs exhibit enhanced caffeine-induced Ca^2+^ release, and older mesenteric arteries from mutant mice display stronger caffeine-induced vasoconstriction. Together, these findings identify RyR2 as a major functional contributor to Ca^2+^ spark and Ca^2+^ wave generation in arterial smooth muscle and demonstrate that increased RyR2 activity can directly influence vascular Ca^2+^ signaling and vascular reactivity.

The relative contribution of the different RyR isoforms to Ca^2+^ spark generation in vascular smooth muscle has remained unclear. Previous genetic studies suggest distinct roles of individual RyR isoforms. Genetic ablation of RyR3 increased Ca^2+^ spark activity in arterial VSMCs [13], whereas conditional deletion of RyR2 abolished Ca^2+^ sparks, indicating that RyR2 is essential for spark generation [14]. Although RyR1 deletion has been reported to impair Ca^2+^ spark activity in vascular preparations such as the aorta, pulmonary arteries, and portal vein [16,17], these vessels differ substantially from resistance-sized arteries where RyR2-dependent mechanisms may predominate. However, Ca^2+^ sparks are generated by clusters of multiple RyR channels within the SR membrane [22]. Therefore, loss-of-function strategies alone cannot unequivocally define the role of individual RyR isoforms, particularly when different isoforms coexist within functionally coupled channel clusters that may be constitutive [14] or dynamically regulated [13]. Loss of Ca^2+^ spark activity after deletion of one isoform may result from disruption of the entire cooperative RyR signaling complex rather than demonstrate that this isoform exclusively drives spark formation. In this context, our RyR2 gain-of-function model provides complementary evidence that increased RyR2 activity alone is sufficient to enhance Ca^2+^ spark generation and promote Ca^2+^ wave propagation in arterial VSMCs.

The RyR2^R4496C^ mutation is known to increase the open probability and sensitivity of the RyR2 channel [19]. The mutation increases the luminal Ca^2+^ dependent activation of RyR2 [23] and enhances RyR2 responsiveness to agonists such as caffeine and β-adrenergic stimulation in cardiomyocytes, thereby promoting spontaneous Ca^2+^ release. The effect of the RyR2^R4496C^ mutation on SR Ca^2+^ content remains controversial, with studies reporting reduced SR Ca^2+^ stores [19,24], unchanged Ca^2+^ contents [25], or reductions only under stress conditions, such as high-frequency stimulation [23] or isoproterenol exposure [26]. However, these studies were performed in cardiomyocytes or HEK293 cells, not in VSMCs. Our findings suggest that enhanced RyR2 sensitivity is a dominant feature of the RyR2^R4496C^ phenotype, reflected by increased Ca^2+^ spark frequency. Pulsed caffeine protocols are well suited to assess SR Ca^2+^ release in RyR2 knockout or loss-of-function models (e.g., [6,14]) but are more difficult to interpret in gain-of-function models, as caffeine-induced [Ca^2+^]_i_ responses and contractions may already approach submaximal or maximal levels, limiting further increases. Thus, these experiments cannot reliably resolve differences in SR Ca^2+^ release or refilling in RyR2^R4496C^ VSMCs. Because Ca^2+^ sparks can recruit neighboring release sites and initiate propagating Ca^2+^ waves [27,28], enhanced RyR2 activity likely facilitates the transition from local Ca^2+^ release events to global intracellular Ca^2+^ signals.

Our experiments further show that Ca^2+^ influx through Ca_v_1.2 L-type Ca^2+^ channels remains the dominant source for Ca^2+^ spark generation in RyR2^R4496C^ VSMCs. Pharmacological inhibition of Ca_v_1.2 L-type channels nearly abolished Ca^2+^ sparks, whereas inhibition of Ca_v_3.2 T-type Ca^2+^ channels or non-selective Ca^2+^-permeable cation channels had no or little effect. These findings indicate that the hierarchy of Ca^2+^ entry mechanisms regulating Ca^2+^ spark activity remains unchanged despite enhanced RyR2 sensitivity caused by the RyR2^R4496C^ mutation. The absence of an inhibitory effect of Ni^2+^ further supports previous observations that T-type Ca^2+^ channels are not major contributors to Ca^2+^ spark generation in older vascular smooth muscle [21]. The greater sensitivity to L-type Ca^2+^ channel blockade may suggest that RyR2^R4496C^-driven Ca^2+^ release relies more strongly on indirect regulation through SR Ca^2+^ refilling mechanisms, potentially reflecting the increased responsiveness of mutant RyR2 channels to luminal SR Ca^2+^ [19].

Several limitations should be considered when interpreting these findings. First, Ca^2+^ spark measurements were performed in isolated VSMCs under controlled experimental conditions and therefore may not fully reproduce the complex regulatory environment of intact resistance arteries. Second, the RyR2^R4496C^ mutation represents a pronounced gain-of-function model that may enhance RyR2-dependent signaling beyond physiological adaptations occurring during vascular ageing or disease. Third, although our data demonstrate a critical role of RyR2 in Ca^2+^ spark generation and Ca^2+^ wave propagation, potential functional interactions between different RyR isoforms within shared channel clusters cannot be excluded. The imaging protocol was optimized to quantify event occurrence and frequency, whereas detailed analyses of spark kinetics that could indicate interactions, including rise time, decay time, duration, and amplitude distributions, were beyond the temporal resolution of the acquisition system. Finally, experiments targeting Ca^2+^ influx pathways were designed to identify predominant regulatory mechanisms rather than fully resolve all contributing pathways. Thus, additional studies will be required to further define the potential involvement of T-type Ca^2+^ channels, TRP channels, and other modulatory mechanisms in RyR-dependent vascular Ca^2+^ signaling.

Our findings provide new insights into mechanisms of vascular dysfunction associated with ageing and metabolic stress [29,30,31]. Ageing, diabetes, and diet-induced metabolic disorders impair vascular regulation and contribute to reduced physiological resilience [32,33]. By identifying RyR2 as a potential major regulator of Ca^2+^ spark generation and Ca^2+^ wave propagation in both young and older VSMCs, our data suggest that altered RyR2-dependent Ca^2+^ signaling may contribute to vascular ageing. As improved management strategies successfully prevent fatal tachyarrhythmias and extend survival, affected individuals may become more susceptible to developing hypertension and other age-associated vascular complications. In particular, age-related changes in Ca^2+^ influx pathways controlling RyR2 activity [21] may disturb vascular Ca^2+^ homeostasis and promote vascular dysfunction in older individuals.

## 4. Materials and Methods

### 4.1. Animals

Experiments were performed in male wild-type mice (RyR2^+^/^+^, C57BL/6N background), used as controls, and in homozygous knock-in mice carrying the RyR2^R4496C^ mutation [34]. This mutation in the RyR2 gene is associated with CPVT, a disorder primarily caused by gain-of-function mutations in RyR2 [18,35]. Animals were studied at two age groups: young (12–16 weeks old, 23–30 g body weight) and older (49–55 weeks old, 32–40 g body weight). All animals were from the Central Service and Research Facility for Laboratory Animals at the University Medicine Greifswald, Germany (ZSFV), Greifswald, Germany. Mice were maintained in a temperature-controlled room (22 °C to 24 °C) on 12–12-h light/ dark cycle. All experimental protocols were performed in accordance with the NIH guidelines (Guide for the Care and Use of Laboratory Animals) and ARRIVE guidelines [36], and approved by institutional animal committees (UMG, OE-017/23 on 13 January 2023).

### 4.2. Isolation of Vascular Smooth Muscle Cells

Mesenteric VSMCs from male RyR2^R4496C^ and litter- or age-matched wild-type RyR2^+/+^ were isolated using a standard enzymatic digestion as previously described [6,14,21]. Briefly, mesenteric arteries were isolated and immediately transferred to cold (4 °C) carbogenated (95% O_2_–5% CO_2_) physiological salt solution (PSS) containing (mM): 119 NaCl, 4.7 KCl, 1.2 KH_2_PO_4_, 25 NaHCO_3_, 1.2 MgSO_4_, 1.6 CaCl_2_, and 11.1 glucose. Arteries were then carefully cleaned of surrounding tissue, cut into small segments, and incubated in Ca^2+^-free Hank’s solution [20] containing (mM): 55 NaCl, 80 sodium glutamate, 5.6 KCl, 2 MgCl_2_, 10 glucose, and 10 HEPES (pH 7.4). The solution was supplemented with 1 mg/ml bovine serum albumin (BSA, Sigma, Taufkirchen, Germany), 0.5 mg/ml papain (Sigma), and 1.0 mg/ml DTT, and tissues were incubated for 30 min at 37 °C. The segments were then incubated in Hank’s solution containing 1 mg/ml collagenase (Sigma; type F and H, mixed at a ratio of 30% and 70%, respectively) and 0.1 mM CaCl_2_ for 17 min at 37 °C. After several washes in Ca^2+^-free Hank’s solution containing 1 mg/ml BSA, cells were dispersed from the arterial segments by gentle trituration. The cell clusters were then incubated with the Ca^2+^ indicator fluo-4 (Acetoxymethyl) AM (8.6 nM, Invitrogen/Thermo Fisher Scientific, Eugene, OR, USA, F23981) and pluronic acid (0.005%, *w*/*v*, Calbiochem, La Jolla, CA, USA) diluted in Ca^2+^-free Hanks’ solution for 50 min at room temperature. After incubation with the Ca^2+^ indicator fluo-4 AM, cells were washed with bath solution containing (mM): 134 NaCl, 6 KCl, 1 MgCl_2_, 2 CaCl_2_, 10 glucose, and 10 HEPES (pH 7.4), and maintained at room temperature for 60 min prior to recordings.

### 4.3. Ca^2+^ Sparks and Waves Imaging Measurements

Images and time-series recordings were acquired using a Zeiss LSM 780 confocal microscope (Zeiss, Oberkochen, Germany) equipped with an Axio Imager and a 40× oil-immersion objective. Clusters of cells were mounted on glass coverslips, and recordings were acquired at 5 Hz (one frame every 200 ms). Fluorescence images were acquired using excitation at 488 nm with an argon laser, and emitted light above 515 nm was collected. The entire area of each cell within the image was analyzed to detect spontaneous Ca^2+^ sparks and Ca^2+^ waves under control conditions and after stimulation with a single pulse of 10 mM caffeine. Following caffeine application, cells were washed with bath solution, and recordings were performed 10 min after stimulation. According to the origin of Ca^2+^ sparks from groups of RyRs [9], Ca^2+^ sparks were defined as local fractional fluorescence increases clearly above the noise level in part of the cell and with a maximum duration of <1 s at half amplitude, whereas Ca^2+^ signals spreading across the whole cell and lasting longer than about 1 s were considered as Ca^2+^ waves [14,37,38]. The frequency of Ca^2+^ sparks and waves was calculated as the number of events per unit scan time. The caffeine-induced peak was measured as previously described [14,21,39]. VSMCs were loaded with the Ca^2+^ indicator fluo-4 AM [20]. Maximal amplitude of caffeine-induced peak fluorescence was normalized by the initial fluorescence value (F/F0) [14,20,21].

### 4.4. Pressure Myography

Third- or fourth-order mesenteric arteries were mounted on a pressure myograph system (Living System Instrumentation, Burlington, VT, USA) and cannulated on two glass micropipettes within a chamber continuously superfused with physiological saline solution (95% O_2_–5% CO_2_; pH 7.4; 37 °C) containing (mM): 119 NaCl, 4.7 KCl, 25 NaHCO_3_, 1.2 KH_2_PO_4_, 1.6 CaCl_2_, 1.2 MgSO_4_, and 11.1 glucose. Arteries were equilibrated at 60 mmHg for 30 min, and contractile responsiveness was assessed by exposure to 60 mM KCl prior to the start of the experiments. After a 15 min recovery period following the KCl test, the inner diameter of the vessel was measured using a Nikon Diaphot microscope (Düsseldorf, Germany) [21,40,41]. Arteries were then stimulated with a single pulse of 10 mM caffeine, and vessel contraction was determined from the change in inner diameter. A second 10 mM caffeine pulse was applied 15 min after the first. The recording system was connected to a personal computer for data acquisition and analysis (HaSoTec, Rostock, Germany) [21,40,41].

### 4.5. Imaging and Statistical Analysis

Analyses were performed using ImageJ (version 1.54d)—Time Series Analyzer V3. Specific lasers and channels for excitation and emission of fluorescence were used, together with the T-PMT channel for a clearer view of cell morphology. Data are expressed as means ± SEM. Statistical differences were assessed by ANOVA, unpaired Student’s *t*-test, and Sidak’s multiple comparisons test. The level of statistical significance was set at *p*< 0.05. Statistical analysis and graphics were performed using GraphPad Prism software (version 7.0).

## 5. Conclusions

In summary, our results demonstrate that RyR2 gain-of-function increases Ca^2+^ spark and Ca^2+^ wave activity in arterial VSMCs and is associated with enhanced spontaneous Ca^2+^ release and altered vascular reactivity. These findings support a role for RyR2 in the regulation of local and propagating Ca^2+^ signals in vascular smooth muscle and suggest that dysregulated RyR2 activity may contribute to altered vascular Ca^2+^ signaling and vascular dysfunction.

## Data Availability

Data described in the manuscript will be made available upon request pending approval by the corresponding author.

## Supplementary Materials

The following supporting information can be downloaded at: https://www.mdpi.com/article/10.3390/ijms27219636/s1.

## Abbreviations

The following abbreviations are used in this manuscript:

RyRs	Ryanodine receptors
VSMCs	Vascular smooth muscle cells
Ca^2+^	Calcium
[Ca^2+^]_i_	Intracellular calcium
SR	Sarcoplasmic reticulum
CPVT	Catecholaminergic polymorphic ventricular tachycardia
AM	Acetoxymethyl
T-PMT	Transmission-photomultiplier tube
