# Supplementary material for: RyR2 Gain-of-Function Increases Ca2+ Spark and Ca2+ Wave Activity in Vascular Smooth Muscle Cells
Source: Int J Mol Sci. 2026 Oct 28;27(21):9636. doi: 10.3390/ijms27219636 (PMC13762476; doi:10.3390/ijms27219636)
Supplement: Supplementary file 1 [file ijms-27-09636-s001.zip › ijms-4633850-supplementary.pdf]

Supplementary Material

RyR2 Gain-of-Function Increases Ca^{2+} Spark and Ca^{2+} Wave Activity in Vascular Smooth Muscle Cells.

Daniele Teixeira Alves [1,2]*, Qidi Zhai [1], Liming Lu [1], Zhugang Chu [1], Dmitry Tsvetkov [1], S. R. Wayne Chen [3], Maik Gollasch [1] and Mario Kassmann [1,2]*

1 Department of Internal Medicine and Geriatrics, University Medicine Greifswald, 17475 Greifswald, Germany
2 DZHK (German Centre for Cardiovascular Research), Partner Site Greifswald, 17475 Greifswald, Germany
3 Libin Cardiovascular Institute of Alberta, Department of Physiology and Pharmacology, University of Calgary, AB T2N 4Z6, Canada

* Correspondence: daniele.teixeiraalves@med.uni-greifswald.de (Alves, D.T.) or mario.kassmann@med.uni-greifswald.de (Kassmann, M); Tel.: +49 3834 864893

1. Supplementary methods

1.1 Pharmacological blockade of direct and indirect RyR2 activation pathways

VSMCs isolated from older mice, as described in Section 2.2 (Isolation of Vascular Smooth Muscle Cells), were incubated for 10 min with blockers of L-type Ca^{2+} channels (Cav1.2; 200 µM Cd^{2+}), T-type Ca^{2+} channels (Cav3.2; 50 µM Ni^{2+}), and TRP channels (100 µM Gd^{3+}), either alone or in combination, to evaluate their effects. The entire area of each cell within the image was analyzed to detect Ca^{2+} sparks under control conditions and after channel blockade. Ca^{2+} spark frequency was calculated as the number of detected events normalized to the total acquisition time.

1.2 Wire myography

First- and second-order mesenteric arteries were isolated, immediately transferred to ice-cold (4°C) PSS oxygenated with 95% O_2/5% CO_2, and dissected into 2-mm arterial segments after careful removal of perivascular adipose and connective tissue. Each ring was positioned between two stainless steel wires (diameter 0.0394 mm) in a 5 ml organ bath of a Mulvany Small Vessel Myograph (DMT 610 M; Danish Myo Technology, Denmark) [1]. The organ bath was filled with PSS. The bath solution was continuously oxygenated with a gas mixture of 95% O2 and 5% CO2, and kept at 37°C (pH 7.4). Rings were placed under tension of 0.3 g [1]. The software Chart8 (AD Instruments Ltd. Spechbach, Germany) was used for data acquisition and display. The rings were precontracted with 60 mmol/l KCl and equilibrated until a stable resting tension was acquired. Tension is expressed as tension/length (mN/mm). Drugs were added to the bath solution [1].

2. Supplementary figures

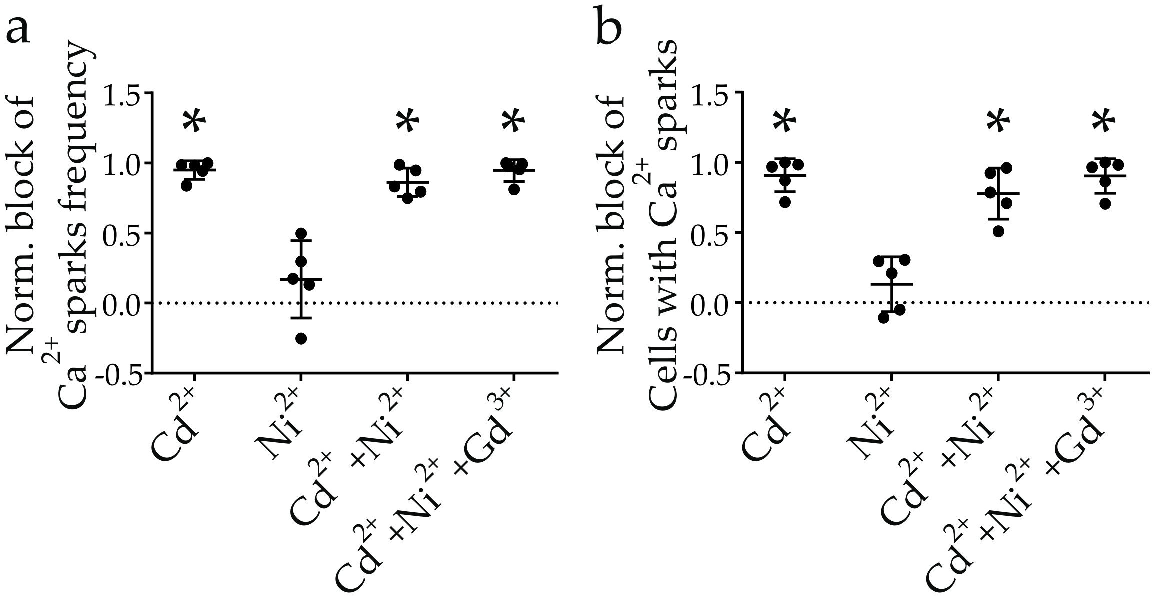

Supplementary Figure S1. Effects of Ca^{2+} channel blockade on Ca^{2+} spark activity in VSMCs from older RyR2^{R4496C} mice. (a) Ca^{2+} spark frequency and (b) fraction of cells producing Ca^{2+} sparks after blockade of $Ca_v1.2$ L-type Ca^{2+} channels (200 µM Cd^{2+}), $Ca_v3.2$ T-type Ca^{2+} channels (50 µM Ni^{2+}), TRP channels (100 µM Gd^{3+}), and combinations. Ca^{2+} spark frequency and fraction of cells were normalized to the respective untreated controls. Values are mean ± SEM. *P<0.05 vs untreated (ANOVA), n=5 mice (RyR2R^{4496C}), 52-154 cells per mouse and group.

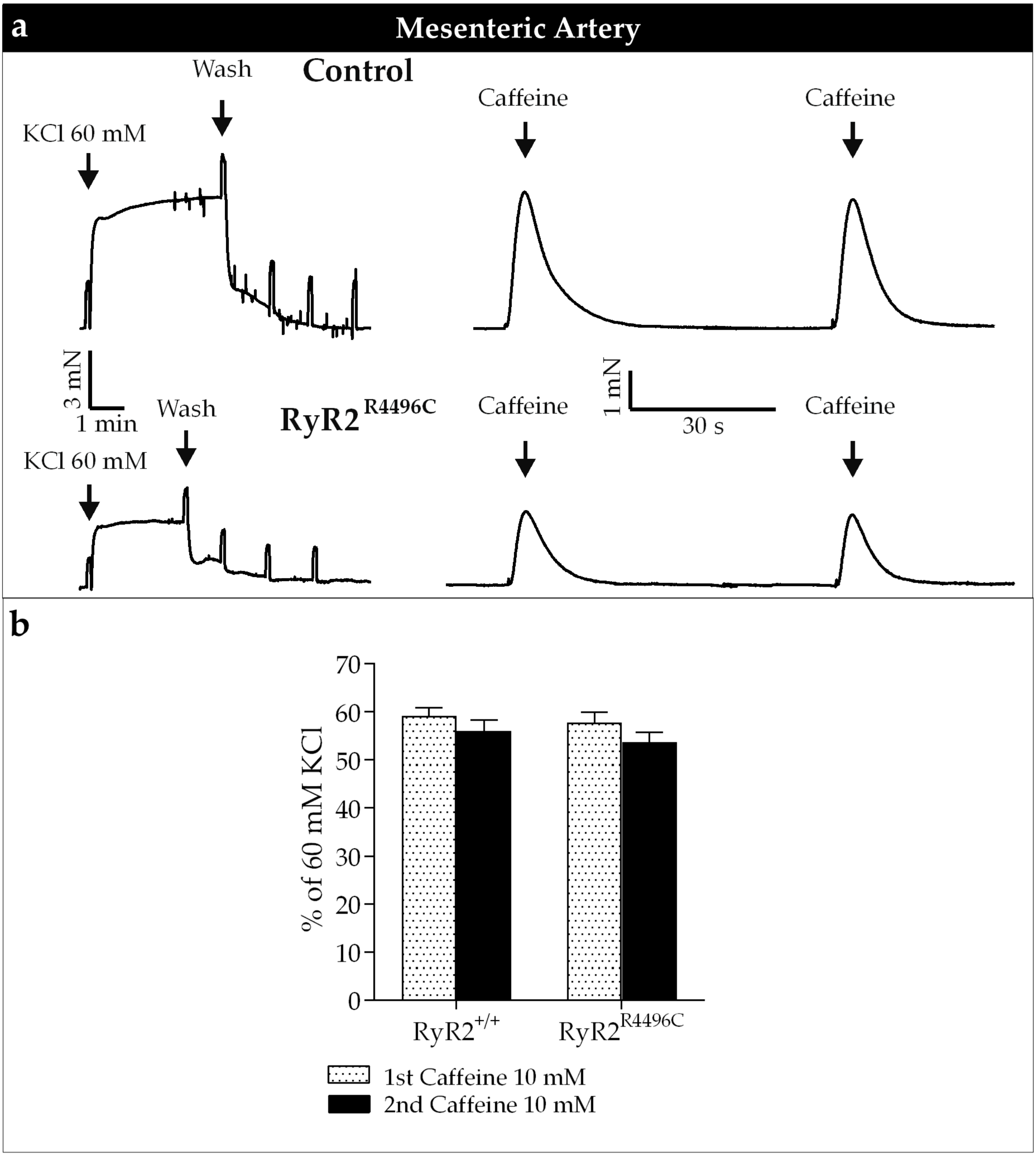

Supplementary Figure S2. RyR2-mediated caffeine-induced vasocontractions in mesenteric arteries from young RyR2^{R4496C} mice. (a) Representative traces of caffeine-induced contractions in mesenteric artery rings from RyR2$^{+/+}$ (upper trace) and RyR2^{R4496C} mice (lower trace) recorded by wire myography. (b) Caffeine-induced vasoconstriction did not differ between young RyR2^{R4496C} and RyR2$^{+/+}$ arteries (Sidak's multiple comparisons test). Values are mean ± SEM. RyR2$^{+/+}$, n = 11 mice; RyR2^{R4496C}, n = 10 mice; 1–4 mesenteric artery rings were analyzed per mouse.

Supplementary References

1. Heinze, C.; Seniuk, A.; Sokolov, M.V.; Huebner, A.K.; Klementowicz, A.E.; Szijarto, I.A.; Schleifenbaum, J.; Vitzthum, H.; Gollasch, M.; Ehmke, H.; et al. Disruption of vascular Ca2+-activated chloride currents lowers blood pressure. *The Journal of clinical investigation* **2014**, *124*, 675–686, doi:10.1172/JCI70025.
